# Underexplored terrain: effects of high priority environmental toxicants on skeletal muscle

**DOI:** 10.1080/10937404.2025.2593824

**Published:** 2025-11-23

**Authors:** Zachary Clemens, Lauren Weaver, Abraham Brown, Jagruti Kosaraju, Aaron Barchowsky, Iva Miljkovic, Amrita Sahu

**Affiliations:** a Department of Physical Medicine and Rehabilitation, University of Pittsburgh School of Medicine, Pittsburgh, PA, USA;; b Department of Epidemiology, University of Pittsburgh School of Public Health, Pittsburgh, PA, USA;; c Mcgowan Institute of Regenerative Medicine, University of Pittsburgh, Pittsburgh, PA, USA;; d Lake Erie College of Osteopathic Medicine, Greensburg, PA, USA;; e Department of Environmental and Occupational Health, University of Pittsburgh School of Public Health, Pittsburgh, PA, USA

**Keywords:** Environmental health, skeletal muscle, Toxicology, environmental epidemiology

## Abstract

In recent decades, evidence has continuously mounted regarding the myriad adverse health effects that environmental exposures exert on human health, yet little attention has been given to skeletal muscle-related outcomes. With its important metabolic, hormonal, and functional properties, skeletal muscle exerts a critical effect on human health and quality of life. The aim of this review was to survey the literature regarding potential impacts of environmental exposures on skeletal muscle health. The focus was on 10 substances atop the Agency for Toxic Substances and Disease Registry’s Substance Priority List including arsenic, lead, mercury, vinyl chloride, polychlorinated biphenyls, benzene, cadmium, benzo(a)pyrene, polycyclic aromatic hydrocarbons, and benzo(b)fluoranthene. In addition, per- and poly-fluoroalkyl compounds were included due to an increasing interest in the field of toxicology. Cell, animal, human, and population studies were all examined to determine toxicant effects on skeletal muscle, though the literature is scarce for many individual agents. Some commonalities, such as effects on mitochondrial function and sexually dimorphic consequences, were observed across compounds. Evidence indicates the need for further investigation of this important topic, with an emphasis on longitudinal large-scale population studies, and investigations which synthesize population and mechanistic research to interrogate causality.

## Introduction

The central dogma of biology where DNA is transcribed into RNA, which is subsequently translated into protein and ultimately shaping phenotype was first proposed by Francis Crick in 1957 and provided a powerful organizing principle for molecular research ever since. However, real-world biology has consistently proven more complex than this linear model suggests. Discoveries such as epigenetic modification and reverse transcription demonstrate that information flow is neither entirely unidirectional nor insulated from environmental influence. These extensions to the central dogma demand an expansion in our consideration of the factors that dictate health and phenotype. Indeed, while not entirely vindicating Watson and Crick’s predecessor, Lamarck, and his famous “giraffe theory” of evolution, the notion that the environment might shape biology across the lifespan and even across generations is increasingly supported by a growing body of evidence. Environmental exposures influence gene expression, mediate RNA translation, modify protein function, and hence affect phenotypic outcomes including the development of environmental-associated diseases (Rappaport and Smith 2010). Certain well-characterized examples of these diseases, such as the relationship between asbestos exposure and mesothelioma, also demonstrate this phenomenon (Selikoff, Churg, and Hammond 1965). In addition, beyond these direct associations, chronic exposure to environmental toxicants of both natural and human origin was linked to a multitude of chronic disease outcomes including cardiovascular, respiratory, and cancer (De Oliveira et al. 2021; Lee et al. 2025; Shkirkova et al. 2020; Utembe and Kamngona 2023). Critical gaps still remain though, especially regarding skeletal muscle where long-term exposure might exert chronic effects on age-related diseases and muscle decline.

Despite its importance, skeletal muscle outcomes are underrepresented in basic, clinical, and population-based toxicology research. This is evidenced by the body of literature reviews considering environmental exposure and muscle health. In the past 30+ years only 4 reviews were published which focused on muscle health and environmental exposures. Most recently was a review on mercury (Hg) (Tam and Rand 2024), preceded by a mini-review in 2024 giving a high-level overview with moderate emphasis on microplastics (Purcaro et al. 2024). Prior to this was a more detailed review of several exposures and skeletal muscle insulin resistance in 2016 (Chehade, Caron, and Aguer 2016). Lastly, in 1993 a review was published largely considering the effects of food and drugs with a brief consideration of toxic exposures on muscle health (Love and Miller 1993). The lack of reviews reflects the apparent absence of primary research in this area. The goal of the present review was to shed light on the importance of skeletal muscle health and its response to environmental exposure by highlighting available studies. The review will provide overviews of the importance of skeletal muscle in maintaining health and its susceptibility to environmental exposures, followed by a scoping review of the primary research in cell, animal, and human models regarding the top 10 items on the Agency for Toxic Substances and Disease Registry’s (ATSDR) Substance Priority List (SPL)—a compilation of toxicants from the CDC deemed to “pose the most significant potential threat to human health” (2022). The review concludes with a synthesis of the key mechanistic and functional findings across these studies and important areas for future exploration.

## The centrality of skeletal muscle to systemic health

Skeletal muscle comprising 30–40% of our body mass is essential for maintaining whole-body health. First, skeletal muscle serves as a key regulator of metabolism and is responsible for approximately one quarter of the body’s resting energy expenditure, nearly as much as the brain (Gallagher et al. 1998). One of its major metabolic functions is to maintain metabolic homeostasis by acting as a reservoir for glucose. Meyer et al. (2002) found that approximately 30% of ingested glucose ultimately is stored in skeletal muscle as glycogen. Skeletal muscle glycogen stores account for the majority of total glycogen storage in the body. Primarily used to provide energy for muscle contraction, depletion of glycogen stores (during activities like exercise) leads to insulin-dependent uptake and storage of glucose from the bloodstream provided by liver mobilization of glycogen stores. In healthy individuals, this inter-organ carbohydrate regulation may prevent ectopic fat accumulation (Argilés et al. 2016; Jensen et al. 2011).

While its role in metabolism has long been recognized, more recently muscle has been demonstrated to also exhibit a unique endocrine role. Skeletal muscle releases unique circulating cytokines, known as “myokines,” with substantial downstream effects on systemic processes (Hoffmann and Weigert 2017). Interleukin-6 (IL6), one of the first myokines identified, increases insulin sensitivity and thus regulates glucose uptake from the circulation (Carey et al. 2006). Interleukin-6 regulatory effects are observed not only in skeletal muscle but also in liver and adipose tissue (Bertholdt et al. 2018). Irisin, another important myokine, also ameliorates insulin resistance (Qiu et al. 2016) and influences lipid metabolism in the liver (Mo et al. 2016). Irisin also mediates the cognitive benefits of exercise (Islam et al. 2021) and supports bone health (Hu et al. 2024). Further, extracellular vesicles (EVs) released by muscles promote muscle regeneration and also represent a potential link between muscle and brain health (K.-Y. Huang et al. 2024; Sahu et al. 2021).

Given the crucial functions skeletal muscle plays in maintaining metabolic homeostasis, it is not surprising that declines in skeletal muscle health often predict or accompany disease or dysfunction throughout the body. Cardiometabolic and skeletal muscle health are tightly linked. Because of its key role in regulating glucose homeostasis, insulin resistance in skeletal muscle precedes even pancreatic β-cell dysfunction in the onset of diabetes (DeFronzo and Tripathy 2009). In addition, increased ectopic fat accumulation in muscle and diminished muscle mass are both independently associated with greater risk of diabetes even in non-obese individuals (Goodpaster et al. 2003; Haines et al. 2022) which was also noted in cardiovascular disease (CVD) (Huynh et al. 2022; Lu et al. 2023). While precise mechanisms are not elucidated, myokines likely play a role, as irisin has been shown to regulate blood pressure (Zhang, Chang et al. 2015). Myokines also appear to possess anti-cancer properties, upregulating certain tumor suppressors and downregulating tumor promoters in several cancer cell lines (Papadopetraki et al. 2022).

Skeletal muscle health is related not only to the onset of various diseases, but also to their outcomes. Skeletal muscle declines and worsening disease pathologies might exacerbate each other, resulting in a downward health spiral. Poor muscle health is related to elevated risk of complications following cardiovascular surgery (Yamashita et al. 2021), along with enhanced risk of mortality rates associated with diabetes (J. Zhao et al. 2025), CVD (Cai et al. 2025), cancer (Cho et al. 2017), and all-causes in adults and the elderly (Cheng et al. 2025; Y. Wang et al. 2023). Further, muscle integrity improves quality of life, reduces risk of falls, and decreases depression symptoms and suicide risk in teenagers (Frank-Wilson et al. 2016; Janssen et al. 2020; Marques et al. 2020; Samuel et al. 2011; N.-P. Yang et al. 2018). This substantial body of evidence is a convincing demonstration of the importance that muscle health plays in regulating whole-body health.

## A brief overview of muscle toxicology

While environmental xenobiotics may enter the body through various points (oral, dermal, inhalation, or mucous membranes), these agents generally access skeletal muscle via the circulatory system. Muscle is a highly vascularized tissue making up a large proportion of the body volume. In addition, muscle vasculature lacks the highly selective membrane of the blood-brain barrier, indicating that most foreign substances might easily transpose from circulation to muscle. Exercise and activity might actually enhance access to muscle cells as more capillaries open and the surface area of exchange between muscle and circulation increases (Tedjasaputra, Bouwsema, and Stickland 2016). Thus, occupational and other environments where individuals are exposed to compounds during exertion might enhance their susceptibility to toxic effects on skeletal muscle (Marfo and Obeng-Gyasi 2024). Further, exertion elevates respiration, which in turn might increase inhalation exposure to certain substances (P. Wu et al. 2024). Some substances gain entry to cells through transporters of essential nutrients, such as lead (Pb) which might use calcium ion (Ca^2+^) transporters due to its similar charge (Rădulescu and Lundgren 2019). Other small nonpolar substances such as benzene might directly cross cell membranes (Odinokov and Ostroumov 2015).

Skeletal muscle is composed of a variety of cells. Muscle fibers are the primary cell type and are responsible for contraction. Given the crucial role of calcium (Ca^2+^) ion transport in initiating and terminating muscle contraction, muscle fibers may be particularly susceptible to substances like Pb which mimic Ca^2+^. By interfering with Ca^2+^ transport these compounds directly inhibit muscle contractile function. Muscle satellite cells (MuSCs) are another important cell type which are activated by muscle injury and drive the regeneration by proliferating, differentiating into muscle progenitor cells (MPCs), and fusing to form new muscle fibers. *In vitro* models commonly use primary MPCs or immortalized MPC cell lines such as C2C12s to study muscle toxicity. While several of these studies are described in more detail below, in general, toxicity in MuSCs and MPCs might lead to increased injury susceptibility, impaired muscle regeneration, and premature muscle aging (Ambrosio et al. 2014; Clemens et al. 2023; S.-E. Wu et al. 2022). Fibroadipogenic progenitor cells (FAPs) also play a role in muscle regeneration and when compromised, may induce fibrosis and/or ectopic fat accumulation in muscle (Anguiano et al. 2020; Biferali et al. 2021). Given the diversity of muscle environment and stressors it is exposed to, there is no single stress-response pathway specific to skeletal muscle. Various shared canonical stress pathways including NRF2 signaling, NF-κB, FoxO, and MAPK are all responsive to environmental stressors (Z. Bao et al. 2022; Padmaja Divya et al. 2015; Wenhong et al. 2025; C. Zhang et al. 2016). However, usefulness of canonical stress pathways for identifying muscle-specific exposures may be limited due to their shared nature throughout different systems.

Cytochrome P450 (CYP) enzymes, the primary drivers of phase I xenobiotic transformation, are responsible for the metabolism of many muscle toxins including most of those presented here (Esteves, Rueff, and Kranendonk 2021). Exceptions are described below. While CYP transformation and phase II conjugation reactions primarily occur in the liver, skeletal muscle was also found to produce some CYP enzymes. However, role of CYP enzymes in metabolizing environmental xenobiotics has not been demonstrated (Hillig et al. 2003). Still, muscles have a confirmed role in the fate of environmental exposures. Metallothioneins (MTs) are a family of proteins which sequester and detoxify many environmental metals. Although MTs are primarily found in liver, skeletal muscle also produces MTs (Durnam and Palmiter 1981). The role of MTs in muscle has important implications for the toxicity of several metals including Hg and cadmium (Cd), which is discussed in the following sections.

## Environmental toxicants of concern and their effects on skeletal muscle health

### Arsenic (As)

Arsenic (As) sits atop the SPL because of its broad toxic effects and risk of exposure. One of the top 20 most prevalent elements in the earth’s crust, this metalloid is naturally found in groundwater and it is estimated that nearly 300 million individuals worldwide have higher than safe levels of As in their drinking water (De Oliveira et al. 2021; Podgorski and Berg 2020). In addition to drinking water, routes of As exposure include ingesting crops grown in contaminated water and soils, and through inhalation of particles in occupational settings, such as smelters and glass manufacturing or environmentally from mine tailings (Formenton et al. 2021; Hughes et al. 2011; Root and Chorover 2023). Once in the body, As is methylated by the enzyme arsenite methyltransferase (AS3MT) using s-adenosylmethionine as a methyl donor. This produces the methylated arsenic species monomethylarsonic acid (MMA) and dimethylarsinic acid (DMA) which might then be conjugated and readily excreted (Watanabe and Hirano 2013). Despite this efficient excretion pathway, As is known to exert significant chronic effects. While As-associated cancers and CVD receive a lot of attention in literature, metalloid effects on skeletal muscle are underexplored. Only in the past decade have investigators considered the influence that environmental exposure to As may have on muscle health.

Animal models demonstrate the impact of As on muscle health. Low dose exposure (100 ppb) of mice lasting 2–5 weeks results in loss of muscle mass, fatigue susceptibility, intramuscular lipid accumulation, and impaired regeneration following injury (Ambrosio et al. 2014; Chen, Chung et al. 2020; Garciafigueroa et al. 2013; C. Zhang et al. 2016). *In vivo* and *ex vivo* studies consistently demonstrate that As increases inflammatory NF-κB signaling (Barchowsky et al. 1996; M. Wei et al. 2016; C. Zhang et al. 2016). In muscles, this disruption induces aberrant extracellular matrix formation, driving muscle satellite cells toward a fibrotic phenotype and suppressing muscle regeneration (Anguiano et al. 2020). However, the inhibition of mitochondrial oxidative signaling, NF-κB activity, and NOTCH-driven cell fate pathways prevents these effects (C. Zhang et al. 2016). Several models demonstrated different presentations of mitochondrial damage and dysfunction following As exposure in muscle. Mitochondria from As-exposed muscle progenitors, myofibroblasts, and muscle fibers exhibit abnormal morphology, producing more reactive oxygen species (ROS), and exhibiting respiratory chain uncoupling (Ambrosio et al. 2014; Anguiano et al. 2020; Clemens et al. 2023). Dysfunctional mitochondrial phenotypes are transmitted to subsequent cellular generations even after the cessation of As exposure (Cheikhi et al. 2019). In addition, mitochondrial epidermal growth factor receptor (EGFR) is phosphorylated in response to As exposure in C2C12 cells (an immortalized mouse myoblast line). Phospho-EGFR associates with complex IV of the mitochondrial respiratory chain, increasing ROS, disrupting mitochondrial membrane potential, and inhibiting C2C12 differentiation (Cheikhi et al. 2020). Interestingly, treatment with the mitochondrial protectants SS-31 (Szeto and Liu 2018) or XJB-5–131 (Javadov et al. 2015) rescues several of the aforementioned phenotypes—reversing As inhibition of muscle regeneration *in vivo* (Anguiano et al. 2020), restoring healthy mitochondrial progeny (Cheikhi et al. 2019), and preventing aberrant EGFR-complex IV binding (Cheikhi et al. 2020). This further demonstrates the crucial role of mitochondria in mediating As effects in skeletal muscle.

**Figure 1.**
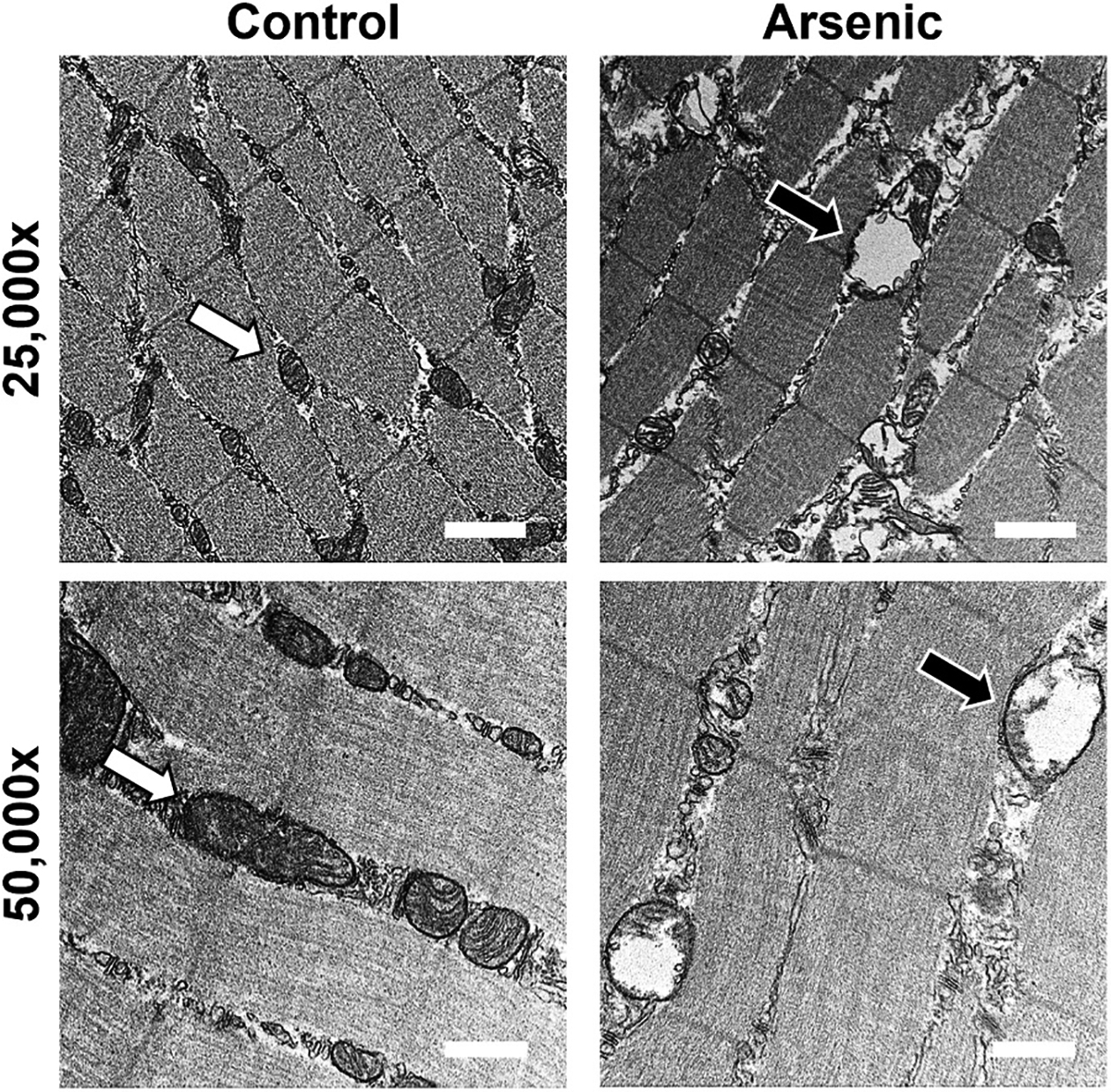
Direct effects of arsenic on skeletal muscle mitochondria. Transmission electron microscopy (TEM) images of the tibialis anterior muscle from female mice given drinking water with or without arsenic (100 μg/L) for 5 weeks (unpublished). Arrows indicate representative mitochondria exhibiting normal (white) versus abnormal (black) morphology. Top scale bars: 1 μm. Bottom scale bar: 500 nm.

Several investigators observed impaired muscle regeneration in As-exposed animals (Ambrosio et al. 2014; Cheikhi et al. 2020; C. Zhang et al. 2016), suggesting that As targets muscle progenitor cells (MPCs). MPCs are typically quiescent in healthy muscles but are activated in response to injury, proliferating, differentiating, and fusing to form new myofibers (Schmidt et al. 2019; Yin, Price, and Rudnicki 2013). Developmental signaling pathways including MAPK, PI3K/Akt, mTOR, and FoxO contribute to MPC-driven regeneration (Elia et al. 2007; García-Prat et al. 2020; Segalés, Perdiguero, and Muñoz-Cánoves 2016; Zhang, Liang et al. 2015). Deficits in MPC function are studied frequently in the context of aging, and age-related declines in mitochondrial function in differentiating MPC which Sahu et al. (2018) postulated to contribute to impaired recovery from injury in a mouse model. However, untangling MPC-specific effects following As exposure presents a significant challenge due to the complexity of the muscle environment. While it may be conceivable that As effect on MPCs are secondary to actions on other tissues, Clemens et al. (2023) utilized a 3D-model of skeletal muscle differentiated from a highly pure population of MPCs to demonstrate that this metalloid in fact directly targets MPCs in a cell-autonomous manner. In addition, published (Ambrosio et al. 2014) and unpublished images (Figure 1) from the same group illustrate that As targets mitochondria in skeletal muscle, resulting in abnormal morphology and significant disruption of the mitochondrial cristae. The effects of As on mitochondrial phenotype were quantified in MPCs and myofibroblasts from As exposed mice (Anguiano et al. 2020; Cheikhi et al. 2019). Taken together these indicate that As directly targets muscle cell mitochondrial function.

Links between As and muscle have been understudied in population-based research. Emerging evidence derived from a few smaller studies suggests that observations in animal and cell models might translate into humans. In Bangladesh, two studies found that As concentrations in water, hair, and nails correlated with increased insulin resistance and reduced muscle mass ascertained from anthropometrically calculated lean body mass and serum creatinine levels (Mondal et al. 2020, *N* = 581 and; Sarker et al. 2021, *N* = 437). In another study Parvez et al. (2011, *N* = 304) observed impaired motor function, measured via the Bruininks-Osterskey test which incorporates fine manual control, manual coordination, body coordination, and strength and agility into a total motor composite score, in children. Globally, it is estimated that 10–15 million As-exposed individuals may suffer from some form of muscle atrophy or sensorimotor impairment (Chakraborti et al. 2003; Mukherjee et al. 2003). Most recently an association with increased adiposity (waist circumference, skin-fold thickness, and serum leptin levels) and reduced calculated lean body mass was detected in the aforementioned Bangladeshi population (Jubayar et al. 2025, *N* = 524). These findings suggest that As induced adverse consequences on muscle health in humans, while also indicating the need for further exploration in different and larger populations.

### Lead (Pb)

Lead (Pb) is a heavy metal with no known physiological function. Anthropogenic activities, including large-scale mining, agriculture, electronic production, and petrochemical usage, are the main source of environmental Pb pollution (Liang and Mao 2015). Exposure occurs primarily through dermal contact (Aljazzar et al. 2021; Mehouel and Fowler 2022), the respiratory system (Aljazzar et al. 2021; Tammone et al. 2021), and by ingesting contaminated food, water, and other materials (Aljazzar et al. 2021; Mehouel and Fowler 2022; Mohammadyan et al. 2019; Monger and Wangdi 2020; Zeinali, Salmani, and Naseri 2019). The method and magnitude of Pb accumulation vary globally (Pemberthy, Padilla, and Peñuela 2021). Several investigators reported dermal Pb exposure detected in artisanal metalworkers and in body painting performers, as well as in some cosmetics and traditional medicine (Niemeier, Maier, and Reichard 2021). Exposure via ingestion may differ based upon cultural dietary habits (Pemberthy, Padilla, and Peñuela 2021; Tammone et al. 2021; Zeinali, Salmani, and Naseri 2019). Because Pb is not readily metabolized or excreted, bioaccumulation and biomagnification result in increasing metal concentration over time and up the food chain. The prevalence of Pb contamination has made it both a concerning environmental contaminant, but also a serious occupational hazard (Koh et al. 2015).

Lead exposure has been linked to many adverse health effects, and exposure is toxic to most body systems. Consequently, Agency for Toxic Substances and Disease Registry (2022) ranked Pb second on the SPL. Bone acts as a reservoir for Pb after exposure, and bone stores of Pb might still be remobilized throughout the body years after exposure (Silbergeld et al. 1993). Infants and children are particularly susceptible as these children absorb more Pb per dosage than adults (Ziegler et al. 1978). Extensive research has been conducted regarding the effects of Pb on various organ systems. While muscle is underrepresented, some effects were reported. The earliest animal study found evidence of fibrosis and muscle degeneration in Rhesus monkeys chronically exposed to Pb over 9 years (Buchheim et al. 1998). More recently, Pb exposure impaired skeletal muscle cell differentiation in C2C12 cells by upregulating HDAC2 expression suggesting an epigenetic mechanism (Gu et al. 2023). Anti-myogenic effects were also demonstrated in mice, which also exhibited marked effects on metabolic enzyme activity in gastrocnemius muscle when exposed to Pb (Das, Pal, and Basu 2020).

Several epidemiological studies considered how Pb exposure affects muscle strength, a proxy for muscle quality. These consistently noted declines in hand dynamometer-measured grip strength with increasing metal burden in children (Nowak-Szczepanska et al. 2022, *N* = 244) and adults (Gbemavo and Bouchard 2021, *N* = 6199). However, adult women were found to be at greater risk than men (Gbemavo and Bouchard 2021). In addition, increasing blood Pb levels predicted elevated frailty scores and enhanced likelihood of mobility limitation in elderly subjects in the U.S. (García-Esquinas et al. 2015, *N* = 5272) as well as in China (Y. Wei et al. 2022, *N* = 1545). While these studies provide compelling evidence of Pb detrimental effects on muscle quality, a gap exists regarding translating mechanistic studies in cell and animal models into humans and testing for associations between metal exposure and muscle in population-based investigations.

### Mercury (Hg)

Mercury (Hg) is a metallic element that is well known for its adverse health effects and has been the cause of multiple health crises. Mercury exists in multiple forms, such as metallic mercury, mercury vapor, and organic mercury. The form of Hg changes how this metal affects the body, with methylmercury (MeHg) being the most common form of exposure (Hong, Kim, and Lee 2012). According to the World Health Organization, human exposure is primarily through the consumption of Hg-contaminated fish (Bernhoft 2012; Branco et al. 2017). Mercury originates in the earth’s crust and through mining, burning coal, or volcanic activity is released into the environment. Inorganic mercury is converted to MeHg by aquatic microorganisms. Organismal MeHg concentrations increase the higher one goes up the food chain in a process known as biomagnification, eventually concentrating in marine predators (and human dietary staples) like tuna and salmon (Bernhoft 2012; Branco et al. 2017). While most individuals contain a trace amount of Hg in their systems, given its aquatic origins, those populations with a seafood-rich diet tend to possess greater metal levels (Hong, Kim, and Lee 2012). Elimination of MeHg first involves demethylation to convert it into its inorganic form. The mechanism for this process in humans is not understood. Following demethylation, inorganic Hg may be excreted. However, excretion rates vary greatly between individuals, an observation which has not been fully explained (Pope and Rand 2021).

Mercury has been well documented to have adverse effects on a wide variety of body systems and tissues (Bernhoft 2012; Branco et al. 2017). However, the impact on skeletal muscle has received little attention until recently. Emerging evidence suggests that skeletal muscle is both a storage location for MeHg and a target of deleterious effects. Mercury exposure might also increase the half-life of metal in the body in a feedback loop which enhances toxicity following chronic exposure. In zebrafish receiving a steady diet of MeHg, skeletal muscle failed to produce metallothionein (MT) 2, (M) a heavy metal binding protein that is involved in detoxification and removal of heavy metals (Gonzalez et al. 2005). One frequently observed effect of MeHg on skeletal muscle is disruption of mitochondrial structure and cell metabolism. In zebrafish, Hg-contaminated skeletal muscle displayed mitochondria of irregular sizes with abnormally shaped cristae and reduced surface area (de Oliveira Ribeiro et al. 2008). A serial analysis of gene expression further supported these findings in zebrafish. Genes involved with mitochondrial metabolism, the electron transport chain, and oxidative stress responses were all downregulated after exposure to Hg (Cambier et al. 2010). These findings are further confirmed in *Drosophila*, where developmental exposure to MeHg resulted in marked changes in genes related to mitochondrial function and vesicle transport specifically in muscle (Beamish et al. 2025).

Mercury also interferes with key metabolic enzymes in skeletal muscle cells. For glycolysis, both human and chicken 3-phosphoglyceromutase are inhibited by Hg, especially in the adult version of the enzyme (Grisolia, Diederich, and Grisolia 1970). In addition, hexokinase, the first enzyme in glycolysis, and phosphofructokinase, the rate-limiting enzyme in glycolysis, are also inhibited by Hg in mouse skeletal muscle cells (Ramírez-Bajo et al. 2014). Mercury also irreversibly binds to glycogen phosphorylase, which is the rate limiting step in converting glycogen into glucose to be used in the skeletal muscle for energy (Xu et al. 2014). In immortalized C2C12 myoblast models, MeHg inhibited differentiation by altering the expression of key myogenic genes including MyoD and MyoG (Culbreth and Rand 2020; Prince and Rand 2018).

In addition to molecular and cellular alterations, several animal studies demonstrated overall effects on skeletal muscle function. In rats exposed to MeHg, clinical symptoms of extremity weakness, muscle wasting, and muscle cramps were observed along with ataxia, accompanied by mitochondrial disturbances when examined histologically (Usuki et al. 1998). However, functional deficits are not always accompanied by histological changes in skeletal muscle. For instance, Rand et al. (2020) reported that in mice exhibiting reduced grip strength when exposed to MeHg, no phenotypical or morphological changes were seen in the skeletal muscle.

Despite the strong body of mechanistic and animal evidence suggesting potential muscle deficits associated with Hg exposure, few human studies have been conducted, mostly specific to occupational or point source exposures. One small-scale (*N* = 6) study of female dental workers, an occupation with high risk of Hg vapor inhalation, found evidence of fiber atrophy in muscle biopsies (Torres et al. 2000). Studies in South Americans exposed to Hg from gold mining industries noted declines in fine motor dexterity assessed via peg turning, pegboard, and finger tapping tests, but not hand grip strength (Dolbec et al. 2000, *N* = 84). In another study in Ecuadorian gold miners Harari et al. (2012, *N* = 309) increased muscle tremors and postural instability were observed as assessed by the CATSYS Neurobehavioral test System. Available population level data shows variability in results. One study of 704 elderly subjects in Korea increased odds of sarcopenia as defined by DXA-derived skeletal muscle mass index was noted associated with elevated blood Hg levels (Yoo et al. 2016). Another study of 6199 adults using the CDC’s National Health and Nutrition Examination Survey (NHANES) data demonstrated no marked association between blood Hg levels and grip strength (Gbemavo and Bouchard 2021). These seemingly contradictory findings indicate the need for more population-level investigation of Hg-initiated effects on muscle health.

### Vinyl chloride (VC)

Vinyl chloride (VC) is a common industrial product used in the synthesis of the polymer polyvinyl chloride (PVC), which is among the most produced global synthetic plastic polymers with widespread applications in automotive parts, consumer goods, construction materials, and medical products (Wang, Liu et al. 2021). Despite PVC’s everyday occurrence, it was not possible to find a single study which specifically considered the effects of this chemical on skeletal muscle. This might be due to the assumption that intact PVC does not possess common route of exposure dermally or orally. Recently though, potential health risks of microplastic exposure have gained widespread attention in the field of environmental toxicology (Fontes et al. 2024; B. Zhao et al. 2024). Environmental microplastics are often the product of the breakdown of larger plastic items such as those composed of PVC. While a full discussion of microplastic exposure and skeletal muscle consequences is beyond the scope of this review, some investigators suggested that microplastics exert adverse effects on muscle health (Chen, Chung et al. 2020; Liu, Zhang et al. 2022; Shengchen et al. 2021), including two which identified products of PVC breakdown as antagonizers of muscle cell function in a C2C12 line and in mice (Chen, Wu et al. 2020; Liu, Zhang et al. 2022). Data indicate that VC and its products cannot be dismissed as a potential source of muscle toxicity. To our knowledge, no apparent human studies investigated associations between VC exposure, or even the broader category of microplastic exposure, and muscle health (Fusagawa et al. 2025).

### Polychlorinated biphenyls (PCBs)

Polychlorinated biphenyls (PCBs) are persistent organic pollutants widely recognized for their adverse health impacts, including significant effects on musculoskeletal tissues. These compounds are derived from industrial processes, particularly as coolants or insulating materials, and are found in various environmental media, including contaminated soil, water, and food (Alcock et al. 1998; Williams et al. 2020). Even though these chemicals are no longer manufactured in the United States, PCBs persist in the environment and continue to pose adverse health risks. Human exposure primarily occurs through contaminated food, air, and occupational contact, with those residing near hazardous waste sites or consuming large amounts of contaminated fish, meat, and dairy products, while inhalation and dermal exposure also contribute to overall risk (Chanmougavally et al. 2023; Williams et al. 2020). Populations at risk include workers in the chemical industry and individuals in regions with high environmental contamination (Chanmougavally et al. 2023).

Pre-clinical studies going back decades demonstrated PCBs ability to induce muscle dysfunction. Early studies in cell lines showed that PCBs inhibited myoblast differentiation by ablating creatine kinase activity, eventually inducing myoblast necrosis (Cannavò et al. 2005; Coletti et al. 2001). One proposed explanation for this is disruption of glucose uptake and metabolism (Mauger et al. 2016). Further, various PCBs were reported to interact directly with skeletal muscle ryanodine receptors such as RyR1, which is responsible for regulating calcium flux during muscle contraction. PCBs induced the receptors to remain stuck in the open state, depleting calcium stores in the sarcoplasmic reticulum and interfering in excitation-contraction coupling (Niknam et al. 2013). Animal studies demonstrated the phenotypic consequences of these effects. In rats, exposure to PCB 126 impaired bone growth and decreased bone length, potentially through disruptions in calcium homeostasis and growth hormone signaling pathways (Williams et al. 2020). A single, high-dose exposure also significantly decreased oxygen consumption, indicating mitochondrial dysfunction consistent with previous findings of disrupted glucose metabolism (Tremblay-Laganière et al. 2019). Another mechanism of PCB-exerted muscle effects is activation of the aryl hydrocarbon receptor (AhR), which leads to altered transcriptional activity and subsequent dysregulation of gene expression affecting both bone and muscle tissues (Williams et al. 2020).

Despite the cell and animal model evidence of muscle-specific PCB effects, findings in humans are scarce. While numerous epidemiological studies identified other pathologies associated with PCB exposure (Montano et al. 2022), few considered musculoskeletal outcomes. Two papers considered the outcome of mass exposure events involving contaminated rice oil in Japan in 1968 and in Taiwan 1978–1979, respectively. Greater PCB exposure was associated with diminished physical function, measured as functional reach and grip strength, in victims of the 1968 event even 40plus years later (Fukushi et al. 2019, *N* = 142). Children of women exposed during the 1978–1979 event exhibited decreased lean muscle mass (Y. L. Guo et al. 1994). At a population level, one recently published study (*N* = 2106) examined sarcopenia risk (assessed via DXA lean mass scans) association with persistent organic pollutants including eight different PCBs demonstrated a negative association between serum PCBs and sarcopenia risk (Tao et al. 2025). However, Tao et al. (2025) acknowledged that this seemingly counterintuitive finding may be influenced by cross-sectional nature of the study, and the fact that organic pollutants tend to be found at higher concentrations in lipid rather than serum. These interesting findings, along with the array of muscle-specific outcomes and mechanisms identified in animal models indicate the need for more consideration of PCB-related effects on human skeletal muscle.

### Benzene

Benzene an organic compound classified as a volatile aromatic hydrocarbon and a volatile organic compound (VOC) is a well-known environmental toxicant. Benzene is a colorless liquid widely used in industrial processes, particularly in the manufacturing of plastics, synthetic fibers, and chemicals leading to occupational exposures linked with increased cancer risk (Smith, Jones, and Smith 2007). In addition, benzene is present in motor vehicle exhaust, cigarette smoke, and contaminated water supplies (Weisel 2010). Globally, millions of individuals are at risk of benzene exposure. Those residing near industrial facilities or workers in the petrochemical and rubber manufacturing industries are particularly vulnerable (Paustenbach, Bass, and Price 1993; Smith, Jones, and Smith 2007; Wallace 1996).

Best known for its role as a leukemic agent, studies of benzene-mediated muscle toxicity in animal models and in humans are extremely limited. In mice, an exposure mimicking cigarette smoke resulted in inflammatory NF-κB activation in muscle, oxidative stress, and activation of pathways linked to the development of insulin resistance (Abplanalp et al. 2018). Recently Scofield et al. (2025) conducting transcriptomic analysis of many mouse tissues following benzene exposure found benzene induced insulin resistance and identified many other differentially expressed genes related to muscle-specific signaling pathways.

While no human studies specifically considered the effects of benzene on muscle. H. Liu et al. (2025) recently reported a cross-sectional study using NHANES data (*N* = 2544) considering associations of VOC metabolites with sarcopenia risk, derived from DXA scans and BMI, noting significant increased risk of sarcopenia with VOC exposure. While not specific to benzene exposure, this study provides evidence for future research regarding benzene and muscle health.

### Cadmium (Cd)

Cadmium (Cd) is a heavy toxic metal that has become a ubiquitous environmental contaminant. While naturally present in small amounts, human activities such as mining, smelting, fossil fuel burning, and other industrial processes have rapidly increased their presence in air and water sources globally (Gao et al. 2024; H. Zhang and Reynolds 2019). Exposure in humans occurs at a number of sources, including through air, tobacco use, and most notably, diet (Wang, Chen et al. 2021; H. Zhang and Reynolds 2019). Certain foods, including cereals and whole grains, rice, seafood, and vegetables, readily accumulate Cd (Gao et al. 2024; Söderholm et al. 2020). Drinking water is also a common source of exposure (Wang et al. 2021b). Cadmium exposure is also an occupational hazard for individuals who work in nickel-cadmium battery production, phosphate fertilizer use and/or production, and waste disposal processes, especially waste incineration (Gao et al. 2024; H. Zhang and Reynolds 2019). Cd binds readily to the protein metallothionein (MT), which may be protective against its toxicity but also greatly extends its half-life in the body as MT is reabsorbed in the kidneys (Nordberg and Nordberg 2022). The steady bioaccumulation of Cd in individuals and ubiquitous presence in the environment attributes to this long biological half-life. It may take decades for Cd to be excreted from the human body, predisposing individuals to years of chronic effects (Ishizaki et al. 2015; Suwazono et al. 2009). Cadmium exposure is linked with many adverse health outcomes including liver injury, kidney damage and urinary dysfunction, osteoporosis and osteomalacia, hypertension, CVD, chronic inflammation, and neurodegenerative conditions. Yet despite the wide array of Cd-associated pathologies, little is known regarding the effects on skeletal muscle.

Cell and animal models following Cd exposure demonstrated metallic ability to induce muscle disease and dysfunction. In zebrafish, metal- induced muscle damage, including fiber excitation-contraction interference, glycogen depletion, and alterations to protein levels and enzymatic activity (Avallone et al. 2015). H. Yang et al. (2022) using zebrafish demonstrated that microplastics increased muscle uptake of Cd at low doses. On a cellular level, Cd exposure was associated with free radical and reactive oxygen species (ROS) generation and altered mitochondrial activity and gene expression (Avallone et al. 2015; C. Chen et al. 2024; Del Águila-Vargas et al. 2020; H. He et al. 2023). Recently Wenhong et al. (2025) using C2C12 myoblasts found that Cd inhibited C2C12 activity via Nrf2-mediated oxidated stress. In mice, drinking water Cd exposure induced accumulation of inflammatory lipids resulting in pro-inflammatory cytokine release resulting in impaired muscle endurance and decreased muscle weight (H. He et al. 2023). Chronic low-level Cd exposure also initiated skeletal muscle mitophagy via the ROS-mediated PINK1/Parkin pathway, leading to inhibition of insulin signaling and insulin resistance in rat skeletal muscle (C. Chen et al. 2024). A similar study in rats noted this same phenomenon, as well as dyslipidemia, inflammation, oxidative stress (da Costa et al. 2024). Increased serum creatine phosphokinase suggested muscle damage as evidenced by decreased muscle fiber diameter and muscle weight (da Costa et al. 2024).

Data regarding Cd effects on muscle in humans is relatively scarce. However, one distribution study in four cadavers showed that Cd accumulated at high amounts in human skeletal muscle (Egger et al. 2019). An epidemiological study using data from NHANES reported a negative relationship between Cd exposure and peak knee extensor strength in middle-aged multi-ethnic adults (M. Wu et al. 2023, *N* = 2052). In older adults, Cd was linked with decreased grip strength, mobility, and frailty (García-Esquinas et al. 2021, *N* = 2549), and decreased walking speed (J. Kim et al. 2018, *N* = 3226). Cadmium exposure was also linked to chronic musculoskeletal pain (CMP) in adults in the United States (Djade et al. 2022, *N* = 4742) and Thailand (La-Up et al. 2018, *N* = 576), but the mechanism remains unknown

### Polycyclic aromatic hydrocarbons (PAHs)

Polycyclic aromatic hydrocarbons (PAHs) are persistent organic pollutants associated with detrimental consequences upon release during incomplete combustion of organic materials such as fossil fuels, wood, and tobacco (Mallah et al. 2022). Major sources of exposure include industrial emissions, vehicular exhaust, cigarette smoke, charred foods, and contaminated water and soil. Humans encounter PAH through inhalation, ingestion, and dermal contact (Mallah et al. 2022). These compounds are a broad class of compounds, however, most research focused on particular PAHs. The ATSDR includes two specific PAHs in the top 10 on the SPL. Findings for each of these are discussed individually below.

#### Benzo[a]pyrene (BaP)

A toxic and carcinogenic PAH, benzo[a]pyrene (BaP), is prevalent in industrial emissions, vehicle exhaust, and tobacco smoke (K.-H. Kim et al. 2013; Li et al. 2022). A significant proportion of exposure also occurs through dietary intake of grilled, charred, or smoked foods (Rengarajan et al. 2015). Industrial workers in aluminum production, coal tar processing, and urban residents exposed to higher levels of atmospheric PAHs are most vulnerable to downstream effects of BaP exposures (Srogi 2007).

Several animal and cell models were used to demonstrate the harmful effects of BaP on skeletal muscle and explore potential mechanisms. An *in vitro* model using C2C12 myoblasts demonstrated that a short exposure resulted in activation of intracellular AhR, resulting in elevated ROS production, inflammation, and impaired differentiation—effects paralleling accelerated sarcopenia (S.-E. Wu et al. 2022). Further, S.-E. Wu et al. (2022) exposed rats *in vivo* to an air pollution mixture containing BaP and observed decreased similar effects on myogenic markers including decreased irisin and increased myostatin. Another mechanism attributed to BaP action in muscle cells may be the ability to disrupt calcium homeostasis by dysregulating RyR1 (Pessah et al. 2001). BaP was also reported to downregulate p38MAPK, MK2, and Hsp70 in C2C12 cells, another pathway by which it inhibits myogenesis (Z. Bao et al. 2022).

Some of these effects have been explored and also confirmed in humans. Mechanistically, exposing primary human muscle-derived progenitor cells to a low dose of BaP in culture resulted in impaired myogenicity, similar to observations detected in C2C12 cells. Further, the MAPK-Akt and AhR pathways were examined and found to play a role in this suppression as inhibiting AhR and NF-κB ameliorated BaP effects (Chiu et al. 2014). Interestingly, inhibiting the estrogen receptor also reversed BaP effects in this study, suggesting gender may partially determine the severity of outcomes due to BaP exposure. One study explored this by considering the correlation between general PAH metabolites and DXA-derived muscle mass (*N* = 2742) and grip strength (*N* = 2462) in the NHANES survey among adults, finding that PAH metabolite levels were inversely associated with muscle mass and function in men, but not women (Sun et al. 2022). Sun et al. (2022) also used a rat model of BaP exposure, finding the same sex-specific effects. These findings represent the only epidemiological data available linking BaP with skeletal muscle deficits, emphasizing the need for further exploration, including consideration of sex-specific effects.

#### Benzo[b]fluoranthene (BbF)

While BaP is perhaps the best-studied PAH, benzo-[b]fluoranthene(BbF) is suggested to be more stable in the environment and thus a better indicator of industrial PAH exposure (Aubin and Farant 2000). Research into health effects of BbF exposure is lacking in general and entirely absent for skeletal muscle-specific effects. The few studies that were conducted demonstrated developmental toxicity and potential atherosclerotic effects via mitochondrial dysfunction (Q. Bao et al. 2023; J. Guo et al. 2020). These may affect skeletal muscle function, but this cannot be stated definitively without further research. To our knowledge there also have been no apparent studies in humans investigating BbF effects on muscle health.

### Per- and poly-fluoroalkyl substances (PFAS)

Per- and poly-fluoroalkyl substances (PFAS) are a group of chemicals frequently found in consumer products due to their water, oil, and dirt-repellant abilities. Due to their distinct, highly stable carbon-fluorine chains, PFAS chemicals have a very long half-life in the environment and thus often described as “forever” chemicals. While this broad group of chemicals has representation on the SPL outside of the top 10, it was decided to consider it here due to the emerging interest in the field of toxicology given its widespread use and understudied health effects.

As the use of PFAS-containing products rises, so does their prevalence in the environment. Recently, exposure to PFAS and products resulting from PFAS degradation was associated with various adverse health effects, making environmental contamination a significant public health concern (Bell et al. 2021). Already the manufacturing of perfluorooctanoic acid (PFOA) and perfluorooctane sulfonic acid (PFOS), two frequently used PFAS species, is prohibited in the United States, though these compounds may still be produced internationally and imported. To replace these chemicals in production, other PFAS derivatives, such as short-chain PFAS, have become popular alternatives. Less is known about the impact alternative PFAS have on health.

Due to the extent of its application, environmental contamination continues to occur through means of industrial emissions, improper waste-water management, and usage of PFAS-containing products (Sunderland et al. 2019). One primary source of dietary exposure is fish and shellfish consumption (Giffard et al. 2022), but cattle exposed to contaminated water also contained higher levels of PFAS, demonstrating another potential entry point into the food web (Mikkonen et al. 2023). While precise toxicokinetics are not well understood, research suggests that PFAS are not readily metabolized by the body and reabsorbed in the kidneys, decreasing excretion and increasing their lifespan in the body (Rosato et al. 2024). Chronic exposure to PFAS in high-risk populations, such as isolated indigenous groups, emphasizes the urgency of addressing PFAS contamination. In one study of 350 Inuit adults in Greenland, 86% of the participants had serum PFAS concentrations in the “severe” risk category (31.9 ng/ml) established by the European Food Safety Authority (EFSA) (Sonne et al. 2023).

High PFAS-derivative levels have been associated with a myriad of adverse health effects. However, little work has been done on skeletal muscle. One study comparing cytotoxicity of PFAS and PFOS compounds in a variety of cell types found that RMS-13 cells (a fibroblast-like cell line originally isolated from human skeletal muscle) were among the most susceptible cell types to PFAS/PFOS effects, with cytotoxicity observed at relatively low concentrations (Solan et al. 2022). A follow-up to this study found that RMS-13 cells displayed increased activities of glutathione peroxidase (GPx) and superoxide dismutase (SOD), both indicators of oxidative stress (Solan et al. 2023). One population study on PFAS and skeletal muscle used NHANES data to find that adults with higher PFAS levels in their serum exhibited lower DXA-derived lean mass index, potentially reflecting decreased muscle mass. This effect was more significant in women, suggesting gender-specific susceptibility (Jia et al. 2024, *N* = 3274). A second population study, mentioned above regarding PCB levels and sarcopenia risk, also considered 4 PFAS compounds, finding that perfluorohexanesulfonate (PFHxS) showed the greatest contribution to risk of sarcopenia of all the compounds considered (Tao et al. 2025). However, separate models considering the joint effect of 4 PFAS together found no marked association between sarcopenia and PFAS. The same limitations mentioned above regarding PCBs, namely the cross-sectional design and potential for adipose tissue distribution, also apply here (Tao et al. 2025). Given the prevalence and persistence of PFAS/PFOS in the environment, these intriguing yet limited findings warrant further exploration.

## Discussion and future directions

### Expanding epidemiological investigations

The 10 substances above represent a small slice of the environmental hazards that exist across the world as well as the gap that exists in the field of environmental muscle toxicology. Characterization of these substances’ mechanistic and functional effects ranges from fairly in-depth (i.e., Hg) to almost non-existent (i.e., VC, benzene, BbF). For substances like Pb, there is sufficient evidence of functional consequences from human data but little mechanistic exploration using cell or animal models, while other substances (As, PCBs, Cd) show the opposite—broader descriptions of mechanisms in pre-clinical models with little connection to human functional outcomes. In either of these cases, connecting epidemiological data with pre-clinical data may prove valuable. The growing field of molecular epidemiology promotes translational research which might aid this goal.

Overall, the greatest gaps in research seem to be at the human and population level. For most of the substances, only one or two studies were conducted in human participants while several had none. Further, most of the studies cited are cross-sectional, utilizing large databases such as NHANES to investigate relationships at a single timepoint. While certainly of great value for their ability to utilize existing data to explore new hypotheses, these studies are limited in the ability to investigate temporal relationships between exposures and outcomes. In addition, investigators cannot examine declines in muscle health over time in subjects which are of great interest for aging populations. Future large-scale human investigations with a longitudinal design, or which simply piggyback on the many ongoing longitudinal studies, might provide valuable evidence regarding environmental effects on muscle health and decline.

Future studies might attempt to answer questions regarding the exposure-response relationship. One challenge of environmental toxicology is ascertaining the relationship between measured exposure (for example, the concentration of a substance in urine or blood), and true exposure (for example, the actual dose of a substance ingested). This might vary based upon the properties of the compound—whether it is rapidly excreted like As versus remaining in the body for decades as is the case for Pb and Cd. Measured exposures may also reflect variation in individual susceptibility to a substance which might be linked to factors such as lifestyle, body composition, and epigenetics. New studies or meta-analyses of available data might help to address some of these important questions in humans.

Many measures of muscle strength and quality are already frequently used in the course of routine neurological exams such as resistance motor function tests, coordination and balance checks, and gait assessment. In addition, there are several relatively simple and easy to implement measurements of muscle function in human studies, including grip and knee extension strength which might also be informative to clinical practice. Applying these common and simple tools to the field of muscle toxicology may enhance future research.

### Sexual dimorphism and potential endocrine disruption from environmental exposure

Another interesting, shared observation is the phenomenon of sexual dimorphism when outcomes were compared between men and women. Lead and PFAS/PFOS showed more significant consequences in women (Gbemavo and Bouchard 2021; Jia et al. 2024), whereas PAHs exerted a greater effect on muscle strength and function in men (Sun et al. 2022). Unfortunately, lack of large-scale epidemiological studies, where male vs. female comparisons are possible, is another common thread shared by many of these substances. Investigations which consider disparate consequences between men and women and examine mechanisms behind these consequences are warranted.

Further, it is unlikely that PFAS and PAHs are the only substances with sexually dimorphic effects. Lead, PCBs, PAHs, and PFAS are frequently cited as endocrine disrupting chemicals (EDCs, Kabir, Rahman, and Rahman 2015). EDCs are a very broad class of compounds whose effects on hormonal signaling have recently been linked with impaired muscle development (Chanmougavally et al. 2023). Recently, a study of 143 adults in Wuhan, China, examined effects of EDC mixtures on muscle mass and grip strength, noting significant negative associations with several dozen EDCs including various PFAS/PFOS species, 3 PAHs, and Pb (K. Huang et al. 2025). The main drawback of this study, and the major difficulty of establishing relationships between EDCs and muscle health, is the element of time. The Wuhan study collected urine samples at three timepoints over just 1 year. EDCs exert developmental effects, as studies on bisphenol A (BPA), the most frequently investigated EDC, demonstrated (Chanmougavally et al. 2023). Thus, the consequences of prenatal or early-life exposure may not be evident for years or decades. While cross-sectional associations may strongly suggest a link between EDCs and muscle health, longitudinal studies across or larges-cale retrospective cohort investigations might be required to uncover and confirm these effects. These studies might likely uncover further sexually dimorphic effects as testosterone and estrogen are two hormones frequently identified as susceptible to EDCs (Jeng 2014).

### Chicken or egg: toxicological interplay between skeletal muscle and other systems

As the preceding section suggests, identifying toxicological mechanisms affecting muscle health is complicated. The present review primarily highlights mechanistic studies which specifically identified processes at play in skeletal muscle cells themselves, generally through the use of *in vitro* primary or immortalized cell models. However, the skeletal, circulatory, nervous, endocrine, and immune systems all intersect in skeletal muscle, making disentangling cause and effect a daunting challenge. Many studies identified toxicological effects of SPL substances on other systems which may result in skeletal muscle declines. A selection of the most relevant is presented here.

Bone health is inextricably linked with muscle health. One prospective study of 1388 men found that bone mass and density, and muscle mass and volume interacted to significantly predict mortality (Kirk et al. 2025; Sonnenschein and Soto 1998). While several substances were associated with declines in bone health, PFAS levels were associated with reduced bone mineral density in a longitudinal study of 441 adolescents and young adults (Beglarian et al. 2024). Cardiovascular and muscle health are also closely related. Not only do toxic substances use the circulatory system to access muscle, but compounds also exert their effects on the vasculature itself (Shkirkova et al. 2020). A systematic review demonstrated that vascular dysfunction is associated with poor muscle architecture and function, possibly due to oxidative stress induced by compromised blood flow to muscle (Dvoretskiy et al. 2020). Arsenic is well-known for its ability to induce endothelial cell remodeling and dysfunction, leading to arterial stiffening as well as increased vascular leakage (Barchowsky et al. 1996; S.-C. Chen and Chen 2008; Straub et al. 2009). Vinyl chloride is another substance which affects the vasculature. A study of 761 men occupationally exposed to VC demonstrated elevated incidence of abnormal capillaries as observed by capillaroscopy (Lopez et al. 2013).

Toxic effects on the central and peripheral nervous systems (CNS and PNS) also deserve comment. The neuromuscular junction (NMJ) is a unique structure where electrical impulses induce chemical signaling which in turn generates muscle contractions. Certain environmental neurotoxins were found to crossover into the PNS and access the CNS via NMJs. Mercury was detected in the cytoplasm of motor neurons and spinal cord motor tracts (Arvidson 1992). This accumulation suggests that neuronal Hg-initiated toxicity occurs through a retrograde axonal transport from the skeletal muscle, through the NMJ, and into the nervous system (Arvidson 1992; Stankovic 2006). Lead also crosses over into the PNS at NMJs (Pamphlett and Bayliss 1992), possibly by accessing the same channels which Ca uses. Cadmium also accesses the CNS via Ca channels, though it has not been shown that this specifically occurs at NMJs (Arruebarrena et al. 2023). While direct evidence of NMJ crossover was not demonstrated for the other reviewed substances, nearly all do cross the blood-brain-barrier (BBB) and thus may influence muscle health indirectly via neurotoxicity. Saunders et al. (2006) in rats noted that oral administration of BaP resulted in accumulation of BaP metabolites in the brain and reduced motor activity was related to increased oxidative stress. However, BaP metabolites were not measured in muscle, thus direct muscle toxicity is not precluded by the study’s findings.

The aforementioned study reflects the challenge of determining directionality of toxicological mechanisms for substances which target multiple organ systems. Is muscle toxicity dependent upon or independent of neurological, cardiovascular, or endocrine effects? Do effects on these different systems act synergistically and if so, as seems likely, what are the consequences? Some investigators attempted to address these questions, such as a study utilizing 3D muscle mimics to identify muscle cell-specific effects of As exposure (Clemens et al. 2023). However, they remain a potent obstacle for the developing field of muscle toxicology and future investigations need to continue to adopt novel methods to approach it.

Challenges in clarifying mechanistic relationships are paralleled by challenges in establishing phenotypic links. As established earlier, declines in muscle health are markedly related to health outcomes such as CVD and cancer. While some temporal relationships have clearly been established, such as skeletal muscle insulin resistance preceding the onset of diabetes (DeFronzo and Tripathy 2009), causal relationships between other muscle-related comorbidities warrant further investigation. For example, Hg, PCBs, and PFAS were all associated with sarcopenia risk (Tao et al. 2025; Yoo et al. 2016). Sarcopenia and CVD are also tightly associated in a bidirectional relationship (N. He et al. 2021; Sasaki and Fukumoto 2022). The difficulty for toxicologists is in establishing whether Hg (for example) causes CVD by way of sarcopenia, or vice versa. As with other gaps in the field, longitudinal studies, which establish the order of incidence for these various comorbidities, might aid in answering these questions.

### Mitochondria as a hub of muscle stress response

By evaluating this body of literature in its entirety, certain common threads shared between toxicants may be discerned. First, mitochondrial dysfunction associated with ROS-promoted oxidative stress are consequences of almost all the considered substances (As, Cd, BaP). Further, Hg and PFAS/PFOS exposure were associated with oxidative stress in muscle, though a definitive link to mitochondrial-ROS and mitochondrial dysfunction was not shown. In addition to their canonical role as the powerhouse of the cell, mitochondria are integral hubs for regulating cellular metabolism and homeostasis. Mitochondria facilitate the synthesis, storage, breakdown, and clearance of protein and lipid metabolic byproducts (Benador et al. 2019; Spinelli and Haigis 2018). Numerous cellular processes rely upon amino acids, enzymes, and fatty acids generated or located in the mitochondria (C.-W. Kim et al. 2010). Further, mitochondria influence cellular stress by modulating redox balance and acting as crucial mediators of regulated cell death (Spinelli and Haigis 2018; Vakifahmetoglu-Norberg, Ouchida, and Norberg 2017). Given their important metabolic and synthetic functions, it is not surprising that several investigators identified mitochondria as targets of toxicity and these components represent an important target for future research and development of therapies to ameliorate the effects of these and other substances.

## Final thoughts

This review was intentionally limited in its scope, covering only the top 10 substances on the SPL plus one extra compound of increasing interest. There are 275 substances on the SPL and certainly many others to consider. For example, the SPL does not include particulate matter (PM) which is frequently studied in the context of air pollution. This is because PM is by nature heterogeneous and so one substance cannot be singled out. However, a recently published review demonstrates that PM were already reported to exert effects on skeletal muscle (Ding, Wan, and Liu 2025). The example of PM also highlights the need for research on the effects of toxicant mixtures, considering potential synergistic effects. Several investigators previously considered possible synergy between Pb, Cd and Hg as these metals affect kidney function, cardiovascular function, and systemic inflammation (Jain 2019; Obeng-Gyasi and Obeng-Gyasi 2024; Wildemann, Weber, and Siciliano 2015). Extension of these studies into muscle, as well as considering other mixtures, are key areas for future research.

## Supplementary Material

Supplemental data for this article can be accessed online at https://doi.org/10.1080/10937404.2025.2593824.

## Data Availability

Data sharing is not applicable to this article as no new data were created or analyzed.
